# Peripheral administration of morphine attenuates postincisional pain by regulating macrophage polarization through COX-2-dependent pathway

**DOI:** 10.1186/1744-8069-10-36

**Published:** 2014-06-14

**Authors:** Kohei Godai, Maiko Hasegawa-Moriyama, Tae Kurimoto, Takayuki Saito, Tomotsugu Yamada, Takahiro Sato, Masayasu Kojima, Yuichi Kanmura

**Affiliations:** 1 Department of Anesthesiology and Critical Care Medicine, Graduate School of Medical and Dental Sciences, Kagoshima University, Kagoshima 890-8520, Japan; 2 Department of Molecular Genetics, Institute of Life Sciences, Kurume University, Kurume, Fukuoka 839-0864, Japan

**Keywords:** Morphine, Postoperative pain, M1/M2 macrophages, Cyclooxygenase-2, Heme oxygenase-1

## Abstract

**Background:**

Macrophage infiltration to inflammatory sites promotes wound repair and may be involved in pain hypersensitivity after surgical incision. We recently reported that the development of hyperalgesia during chronic inflammation is regulated by macrophage polarity, often referred to as proinflammatory (M1) or anti-inflammatory (M2) macrophages. Although opioids such as morphine are known to alter the inflammatory milieu of incisional wounds through interactions with immunocytes, the macrophage-mediated effects of morphine on the development of postincisional pain have not been well investigated. In this study, we examined how morphine alters pain hypersensitivity through phenotypic shifts in local macrophages during the course of incision-induced inflammation.

**Results:**

Local administration of morphine in the early phase, but not in the late phase alleviated mechanical hyperalgesia, and this effect was reversed by clodronate-induced peripheral depletion of local macrophages. At the morphine-injected incisional sites, the number of pro-inflammatory F4/80^+^iNOS^+^M1 macrophages was decreased during the course of pain development whereas increased infiltration of wound healing F4/80^+^CD206^+^M2 macrophages was observed during the early phase. Morphine increased the gene expression of endogenous opioid, proenkephalin, and decreased the pronociceptive cytokine, interleukin-1β. Heme oxygenase (HO)-1 promotes the differentiation of macrophages to the M2 phenotype. An inhibitor of HO-1, tin protoporphyrin reversed morphine-induced analgesic effects and the changes in macrophage phenotype. However, local expression levels of HO-1 were not altered by morphine. Conversely, cyclooxygenase (COX)-2, primarily produced from peripheral macrophages in acute inflammation states, was up-regulated in the early phase at morphine-injected sites. In addition, the analgesic effects and a phenotype switching of infiltrated macrophages by morphine was reversed by local administration of a COX inhibitor, indomethacin.

**Conclusions:**

Local administration of morphine alleviated the development of postincisional pain, possibly by altering macrophage polarity at the incisional sites. A morphine-induced shift in macrophage phenotype may be mediated by a COX-2-dependent mechanism. Therefore, μ-opioid receptor signaling in macrophages may be a potential therapeutic target during the early phase of postincisional pain development.

## Background

Peripheral neuroimmune interactions play an important role in the development of pain hypersensitivity and in the process of wound healing after surgery. Macrophages, predominately activated during the early stage of the postoperative periods, eliminate necrotic tissue and protect the wound from post-surgical infection by increasing their phagocytic activity [1].

Macrophages can acquire distinct functional phenotypes depending on their microenvironment, such as is present at inflamed sites. Two well-established polarized macrophage phenotypes are proinflammatory (M1) and wound healing (M2) macrophages. M1 macrophages produce high levels of toxic intermediates associated with increased phagocytic activity and pro-nociceptive mediators, such as inducible NO synthase (iNOS) and cyclooxygenase (COX)-2 [2], whereas M2 macrophages display homeostatic functions linked to wound healing [3]. Mice lacking CCR2, a marker for the M1 phenotype and a receptor for macrophage-specific chemo-attractant macrophage chemoattractant protein-1, showed impaired inflammatory pain development and decreased macrophage infiltration to complete Freund’s adjuvant (CFA)-induced sites of inflammation [4], suggesting that M1 macrophages exacerbate the development of hyperalgesia after inflammation. The influx of M2 macrophages in the late phase is preceded by an influx of M1 macrophages [5]. The phenotypic shift in macrophages toward an M2 phenotype is predominantly promoted by heme oxygenase (HO)-1, a stress-responsive enzyme with potent antioxidant and anti-inflammatory activities that is induced immediately after incision and that has antihyperalgesic effects against inflammatory pain after formalin injection [6-8]. We recently demonstrated that the activation of peroxisome proliferator-activated receptor (PPAR)γ signaling promotes macrophage polarization towards the M2 phenotype through an HO-1-dependent pathway, attenuating the development of CFA-induced inflammatory pain [6]. In addition, PPARγ agonist alleviated postincisonal pain by regulating macrophage phenotype [9]. Therefore, the balance between these two subsets of macrophages plays a crucial role in regulating the inflammation and pain development processes.

Peripherally-applied morphine can attenuate inflammatory pain induced by carrageenan [10] and CFA [11]. Morphine regulates the production of neurotransmitters involved in nociception, such as substance P and iNOS, by phagocytes at inflamed sites [12,13]. Recently, the peripheral actions of opioids on immune cells during the course of postoperative wound repair have been suggested. It was reported that morphine inhibited the monocyte-macrophage conversion phase, resulting in delayed migration of monocytes at the sites of injury [14]. Peripheral administration of morphine suppressed the phagocytic activity of macrophages and promoted apoptosis by an HO-1-dependent mechanism [15,16]. These data suggest that morphine might regulate peripheral sensitization through local neuro-inflammatory processes, particularly macrophages.

In closing wounds that were locally treated with morphine sulfate, macrophage infiltration was decreased in the early phase but increased in the late phase of the wound healing process [17]. In addition, acute morphine administration reduced peri-incisional expression of proinflammatory cytokines such as interleukin (IL)-1β and tumor necrosis factor (TNF)-α [18], which are major nociceptive mediators produced by M1 macrophages [19]. Therefore, these reports support our hypothesis that morphine alters the M1/M2 balance in incised sites during the postoperative period.

Although acute peripheral actions of morphine have been suggested, the macrophage-mediated effects of morphine on the development of postincisional pain have not been well investigated. Therefore, we first examined whether repeated administrations of morphine to incised sites had analgesic effects, and then evaluated whether the effects of morphine were mediated by a shift in macrophage polarity at the incision sites.

## Results and discussion

### Local administration of morphine in the early phase of postincisional pain development ameliorates mechanical hyperalgesia

**Figure 1:**
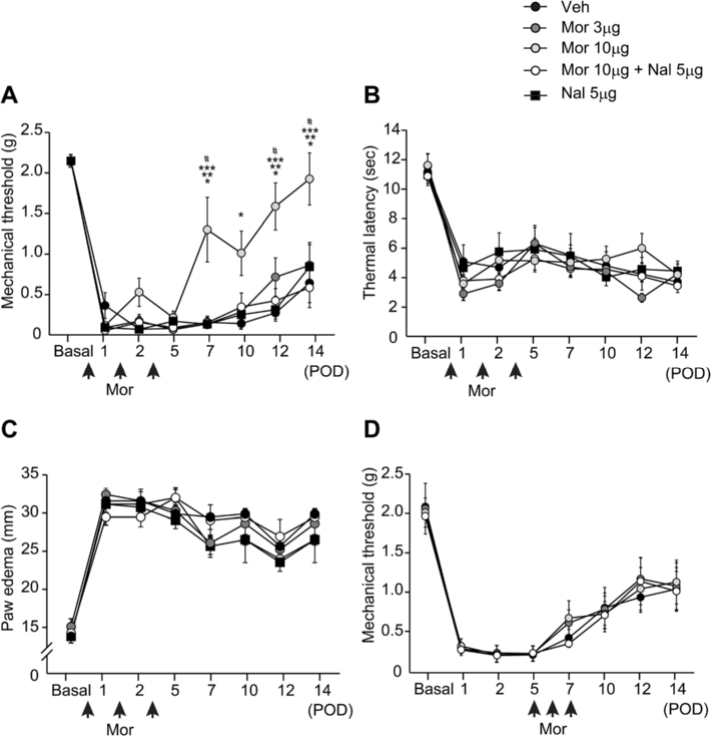
**Local administration of morphine in the early, but not the late phase, alleviates hypersensitivity to mechanical stimuli.** Morphine (3 μg or 10 μg) and/or naloxone (5 μg) were injected into the incisional sites during the early phase (1 h after incision and on PODs 1 and 2) or late phase (PODs 5, 6, and 7). The withdrawal threshold to mechanical stimuli **(A)**, withdrawal latency to thermal stimuli **(B)**, and paw edema **(C)** were examined in groups, receiving morphine in the early phase. The withdrawal threshold to mechanical stimuli was not altered by morphine administered in the late phase **(D)**. *P < 0.001, Mor 10 μg compared with Veh, **P < 0.001, Mor 10 μg compared with Mor 3 μg, ***P < 0.0001, Mor 10 μg compared with (Mor 10 μg + Nal 5 μg), ^#^*P* < 0.001, Mor 10 μg compared with Nal 5 μg (two-way ANOVA followed by Bonferroni *post-hoc* testing). Data are mean ± SEM (Figure 1A-C, n = 8 for each group, Figure 1D, n = 5 for each group). Veh; vehicle, Mor; morphine, Nal; naloxone.

To evaluate the effects of locally administered morphine on the development of postincisional hyperalgesia, morphine was injected into the incised sites daily for 3 days during the early phase (an hour after the procedure and then on postoperative days [POD] 1 and 2) or during the late phase (on PODs 5, 6, and 7). Hyperalgesia to mechanical stimuli was attenuated on PODs 7, 12, and 14 in mice that received morphine in the early phase. This attenuation was reversed to the level of vehicle-injected mice by coadministration of a non-selective opioid receptor antagonist, naloxone (Figure 1A, two-way analysis of variance (ANOVA): F(4, 245) = 12.23, *P* < 0.0001). In contrast to the effects of morphine on mechanical stimuli, morphine had no effect on withdrawal latency to thermal stimuli (Figure 1B, two-way ANOVA: F(4, 245) = 1.15, *P* = 0.35) or on paw edema (Figure 1C, two-way ANOVA: F(4, 245) = 1.35, *P* = 0.27). Morphine administered during the late phase did not elevate the mechanical threshold (Figure 1D, two-way ANOVA: F(3, 112) = 0.20, *P* = 0.89), suggesting that local morphine administration during the early phase, but not the late phase, increased the mechanical threshold.

### Long-lasting analgesic effects of morphine on mechanical hyperalgesia are mediated by local macrophages

**Figure 2:**
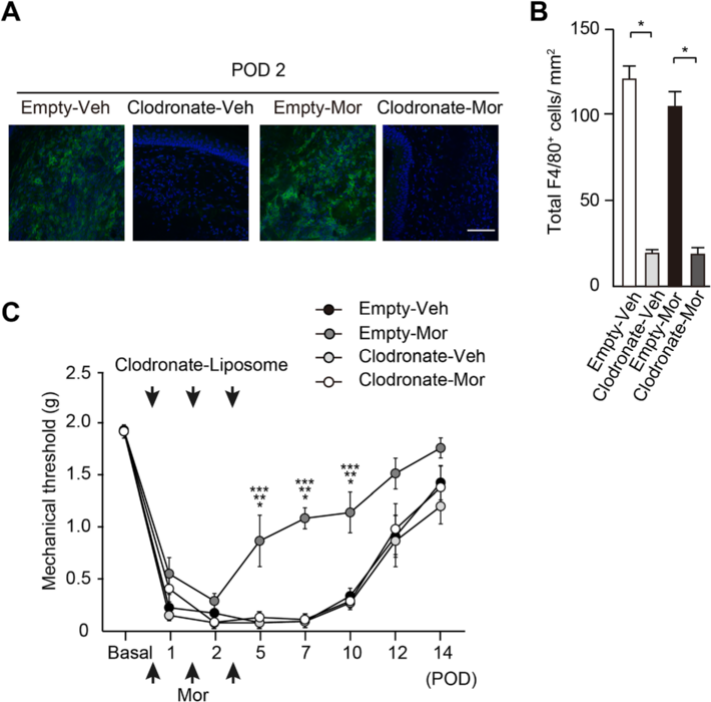
**Depletion of local macrophages resulted in decreased analgesic effects of morphine. (A)** Macrophage depletion after intraplantar injection of 10 μL clodronate liposomes was evaluated by immunostaining for local F4/80^+^ macrophages. Green: F4/80; blue: DAPI. Scale bar: 50 μm. **(B)** The number of F4/80^+^ macrophages was reduced by clodronate liposomes. *P < 0.01 (two-way ANOVA followed by Bonferroni *post-hoc* testing). Data are mean ± SEM (n = 6 for each group). **(C)** The elevated withdrawal threshold to mechanical stimuli induced by morphine was reversed by clodronate liposomes. *P < 0.05, Empty-Mor compared with Empty-Veh, **P < 0.05, Empty-Mor compared with Clodronate-Veh, ***P < 0.05, Empty-Mor compared with Clodronate-Mor (two-way ANOVA followed by Bonferroni *post-hoc* testing). Data are mean ± SEM (n = 8 for each group). Empty-Veh; empty liposomes 10 μL + vehicle (PBS), Clodronate-Veh; clodronate liposomes10μL + vehicle (PBS), Empty-Mor; empty liposomes 10 μL + morphine 10 μg, Clodronate-Mor; clodronate liposomes 10 μL + morphine 10 μg.

We next examined whether morphine exerted its analgesic effects via infiltrated macrophages. To deplete local macrophages accumulated at the incised sites, clodronate liposomes were directly injected an hour after incision and on PODs 1 and 2. Macrophage depletion was evaluated by immunostaining cells with pan-macrophage marker, F4/80^+^ and 4′,6-diamidino-2-phenylindole (DAPI) on POD2 (Figure 2A). The number of F4/80^+^ cells was decreased following intraplantar injection of clodronate liposomes in both vehicle- and morphine-treated paws (Figure 2B, one-way ANOVA: F(3, 23) = 72.60, *P* < 0.0001) although macrophage depletion alone did not change the sensitivity to mechanical stimuli (Figure 2C). However, the analgesic effects of morphine on mechanical stimuli were reversed by depletion of infiltrated macrophages (two-way ANOVA: F(3, 196) = 18.82, *P* < 0.0001). The numbers of F4/80^+^ cells in empty control-injected paws were not altered by morphine. Therefore, macrophages with a specific phenotype increased by the administration of morphine but not by vehicle in the early phase, may contribute to the analgesic effects of morphine. Taken together with the results that morphine administered in the late phase had little effect on the mechanical threshold (Figure 1D), this suggests that morphine likely acts on macrophages accumulating in the early phase. Macrophages are recruited and activated at local sites within 48 hours after the initiation of inflammation [20,21]. During the course of pain development, proinflammatory M1 macrophages accumulate immediately, followed by a phenotype shift to wound healing M2 macrophages in the late phase [5,6]. Therefore, we hypothesized that morphine can attenuate postincisional pain by regulating phenotype switching of macrophages in the early phase.

### Morphine alters macrophage polarity in incision-induced local inflammation

**Figure 3:**
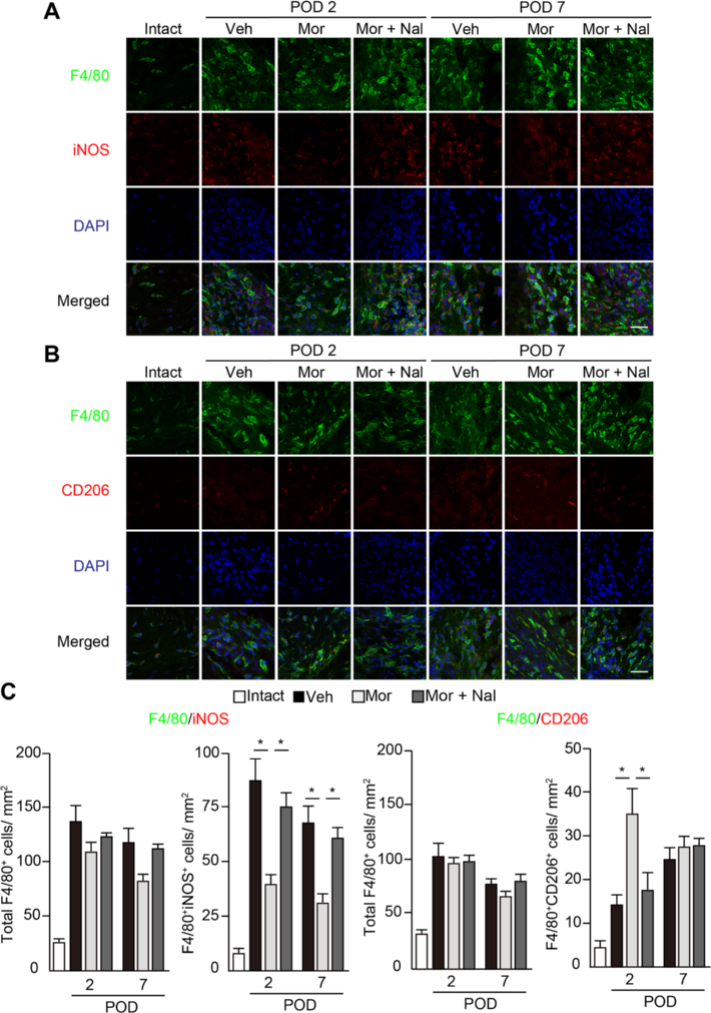
**Morphine induces phenotype shifts of local macrophages.** The infiltration of F4/80^+^iNOS^+^ M1 macrophages **(A)** and F4/80^+^CD206^+^ M2 macrophages **(B)** was evaluated on PODs 2 and 7. **(C)** The numbers of total F4/80, F4/80^+^iNOS^+^, and F4/80^+^CD206^+^ macrophages were counted. Scale bar, 25 μm. *P < 0.05 (two-way ANOVA followed by Bonferroni *post-hoc* testing). Each column represents mean ± SEM (n = 6 for each group). Veh; vehicle (PBS), Mor; morphine 10 μg, Nal; naloxone 5 μg.

To determine the macrophage phenotype at morphine-injected incised sites, numbers of macrophages were counted by immunostaining with F4/80, DAPI and either iNOS, an M1-specific marker (Figure 3A), or CD206, an M2-specific marker (Figure 3B). Consistent with a previous report showing morphine inhibited the induction of NOS in macrophage cell line [22], the number of F4/80^+^iNOS^+^ M1 macrophages was significantly decreased by administration of morphine compared with vehicle, and this effect was reversed by coadministration of naloxone on both PODs 2 and 7 (Figure 3C, two-way ANOVA, F (2, 30) = 22.65, *P* < 0.0001). Differences in the total number of F4/80^+^ macrophages were not statistically significant. Consistent with decreased numbers of M1 macrophages, the number of F4/80^+^CD206^+^ M2 macrophages was markedly increased by morphine compared with vehicle or coadministration of naloxone on POD2 (Figure 3C, two-way ANOVA, F(2, 30) = 5.74, *P* = 0.008) though this difference was not apparent on POD7.

**Figure 4:**
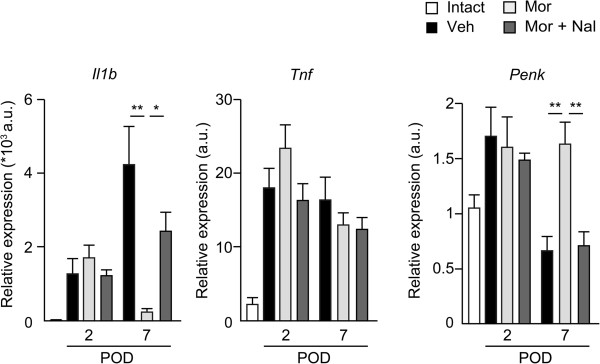
**Morphine decreases the expression of pro-nociceptive cytokine, IL-1β and increased the expression of endogenous opioid, enkephalin.** The expression levels of *Penk* were increased at morphine-treated sites. The expression levels of *Il1b* were reduced at morphine-treated sites, whereas morphine did not alter the expression levels of *Tnf*. *P < 0.05, **P < 0.01 (two-way ANOVA followed by Bonferroni *post-hoc* testing). Data are mean ± SEM (n = 8 for each group). Veh; vehicle (PBS), Mor; morphine 10 μg, Nal; naloxone 5 μg, *Penk*; preproenkephalin, *Tnf*; tumor necrosis factor, *Il1b*; interleukin-1β.

To assess further the effects of morphine on the incision-induced inflammation, we evaluated gene expression of pro-nociceptive cytokines, IL-1β and TNF-α. Consistent with a decrease in iNOS^+^ M1 macrophages at morphine-treated incisional sites, IL-1β mRNA (*Il1β*) was markedly decreased at morphine-treated sites on POD7 (Figure 4, two-way ANOVA, F(2, 42) = 5.73, *P* = 0.006). However, changes in the levels of TNF-α mRNA (*Tnf*) were not significant (Figure 4, two-way ANOVA, F(2, 42) = 1.30, *P* = 0.28). We previously reported that an endogenous opioid, enkephalin, but not β-endorphin or dynorphin produced by M2 macrophages might attenuate CFA-induced inflammatory pain [6]. To investigate whether the analgesic effects of morphine were due to increased production of enkephalin, gene expression of proenkephalin (*Penk*) was measured. The expression levels of *Penk* were 2.5-fold higher in morphine-treated sites compared with vehicle-treated sites on POD7 (Figure 4, two-way ANOVA, F(2, 42) = 4.12, *P* = 0.023). These data suggest that the macrophage-mediated analgesic effects by morphine might be due to the morphine-induced decrease of the pro-nociceptive cytokine, IL-1*β,* or an increase in enkephalin through the phenotype shift of macrophages in the early phase.

### Morphine attenuates mechanical hyperalgesia by acting downstream of HO-1

**Figure 5:**
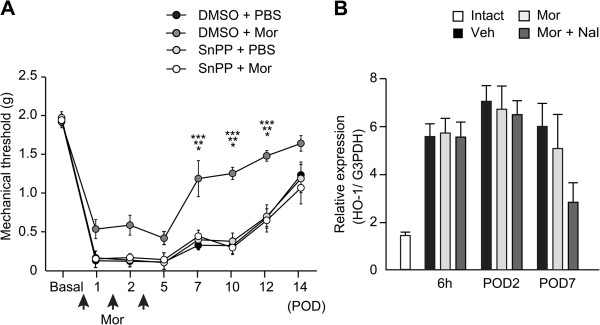
**HO-1 inhibitor SnPP reverses the attenuation of mechanical hyperalgesia by morphine. (A)** The withdrawal threshold to mechanical stimuli was examined. *P < 0.001, (DMSO + Mor) compared with (DMSO + PBS), **P < 0.001, (DMSO + Mor) compared with (SnPP + PBS), ***P < 0.001, (DMSO + Mor) compared with (DMSO + Mor) (two-way ANOVA followed by Bonferroni *post hoc* testing). Data are mean ± SEM (n = 7 for each group). **(B)** Morphine did not alter expression levels of *Hmox-1*. *P < 0.05. Each bar represents mean ± SEM (n = 6 for each) Mor; morphine 10 μg, SnPP; tin protoporphyrin 400 nmol.

**Figure 6:**
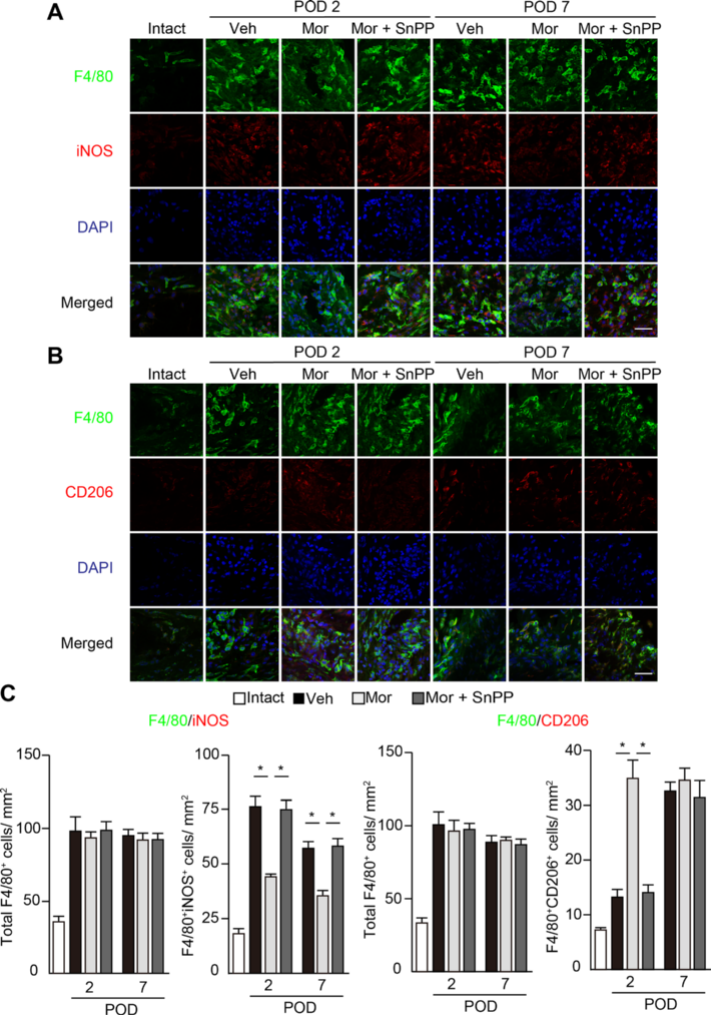
**HO-1 inhibitor SnPP blocks morphine-induced phenotype shift of local macrophages.** The infiltration of F4/80^+^iNOS^+^ M1 macrophages **(A)** and F4/80^+^CD206^+^ M2 macrophages **(B)** were evaluated on PODs 2 and 7. **(C)** The numbers of total F4/80, F4/80^+^iNOS^+^, and F4/80^+^CD206^+^ macrophages were counted. Scale bar, 25 μm. *P < 0.001 (two-way ANOVA followed by Bonferroni *post-hoc* testing). Each column represents mean ± SEM (n = 6 for each group). Veh; vehicle (PBS + DMSO), Mor; morphine 10 μg, SnPP; tin protoporphyrin 400 nmol.

Induction of HO-1 promoted a shift in phenotype to M2 macrophages and the wound healing process [23]. We previously demonstrated that HO-1 was dominantly expressed by infiltrated macrophages in the early phase of CFA-induced inflammation [6]. Because morphine-induced apoptosis of macrophages is inhibited by HO-1 inhibitors, and HO-1 inducers enhanced the effects of μ-opioid receptors during neuropathic pain [24], we examined the involvement of HO-1 in morphine-induced analgesia and the phenotypic shift of macrophages. An HO-1 inhibitor, tin protoporphyrin (SnPP), was coadministered with 10 μg of morphine 1 hour after the procedure and on PODs 1 and 2. Consistent with previous reports, intraplantar injection of SnPP with morphine significantly decreased the mechanical threshold compared with a single injection of morphine (Figure 5A, two-way ANOVA: F (3, 168) = 30.67, *P* < 0.0001). Plantar incision induced a 3.4-fold increase in HO-1 mRNA (*Hmox-1*) expression in vehicle-treated hind-paws 6 hours after incision compared with intact hind-paws. However, morphine did not change the expression level of *Hmox-1* compared with vehicle controls or morphine coadministered with naloxone (Figure 5B, two-way ANOVA: F (2, 30) = 1.10, *P* = 0.34). In addition, SnPP reversed the decrease in M1 macrophages and the increase in M2 macrophages induced by morphine (Figure 6, F4/80^+^iNOS^+^ M1 macrophages, two-way ANOVA: F (2, 30) = 40.53, *P* < 0.0001, F4/80^+^CD206^+^ M2 macrophages, two-way ANOVA: F (2, 30) = 17.90, *P* < 0.0001). These data suggest that HO-1 is essential for morphine-induced analgesic effects and phenotype switch of macrophages. However, since HO-1 expression was not altered by morphine, it is likely that μ-opioid receptor signaling may be down-stream of HO-1, or HO-1 induction itself is independent of μ-opioid receptor signaling in macrophages.

### Analgesic effects of morphine on mechanical stimuli was reversed by COX inhibitor

**Figure 7:**
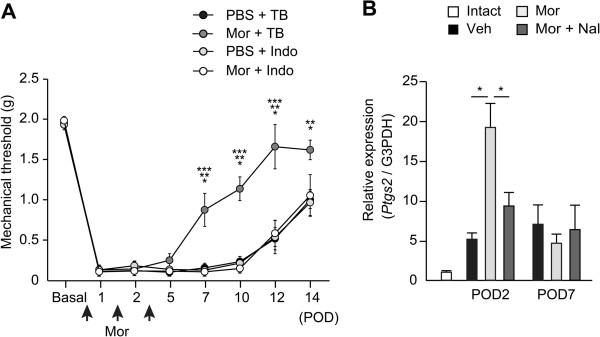
**COX inhibitor, indomethacin reverses morphine-induced elevation of mechanical threshold. (A)** The withdrawal threshold to mechanical stimuli was examined. *P < 0.05, (Mor + TB) compared with (PBS + TB), **P < 0.05, (Mor + TB) compared with (PBS + Indo), ***P < 0.01, (Mor + TB) compared with (Mor + Indo) (two-way ANOVA followed by Bonferroni *post-hoc* testing). Data are mean ± SEM (n = 8 for each group). **(B)** Morphine increased the expression of *Ptgs2* at the incised sites. *P < 0.05 (two-way ANOVA followed by Bonferroni *post-hoc* testing). Each bar represents mean ± SEM (n = 6–9 for each group). TB; tris buffer, Mor; morphine 10 μg, Indo; indomethacin 50 μg, Veh; vehicle (PBS), Nal; naloxone 5 μg.

Prostaglandin E2 (PGE2), a key mediator during acute inflammation, contributed to hyperalgesia by promoting sensory neuron hyperexcitability [25]. The majority of PGE2 synthesis upon initiation of the inflammatory response is mediated by a COX-2-dependent pathway in macrophages, whereas COX-1 is constitutively expressed in nearly all cell types for house-keeping functions. It was reported that morphine directly enhanced the release of arachidonic acid and its metabolites in murine peritoneal macrophages [26]. The release of PGE2 was increased from peritoneal macrophages isolated from rats after receiving morphine for 4 days [27]. To examine the involvement of COX-2 in morphine-induced analgesia in post-incisional pain development, the nonselective COX inhibitor indomethacin was locally administered with morphine 1 hour after the procedure and on PODs 1 and 2. Indomethacin administered with morphine in the early phase decreased the mechanical threshold compared with a single injection of morphine after POD7 (Figure 7A, two-way ANOVA: F(3, 196) = 24.23, *P* < 0.0001). Plantar incision induced a 5.1-fold increase in the expression of COX-2 mRNA, prostaglandin-endoperoxide synthase (*Ptgs*)*2* in vehicle-treated hindpaws on POD2 compared with intact hindpaws (Figure 7B, two-way ANOVA: F(2, 41) = 3.30, *P* < 0.05). Consistent with a previous report that morphine increased PGE2 release from macrophages [27], morphine markedly increased the expression of *Ptgs2,* with the maximum level occurring on POD2 (19.1 fold), which was reversed by naloxone. Therefore, morphine may attenuate post-incisional hyperalgesia by regulating COX-2/PGE2-dependent mechanisms in macrophages.

### Morphine alters the phenotype of local macrophages through COX-2-dependent mechanism

**Figure 8:**
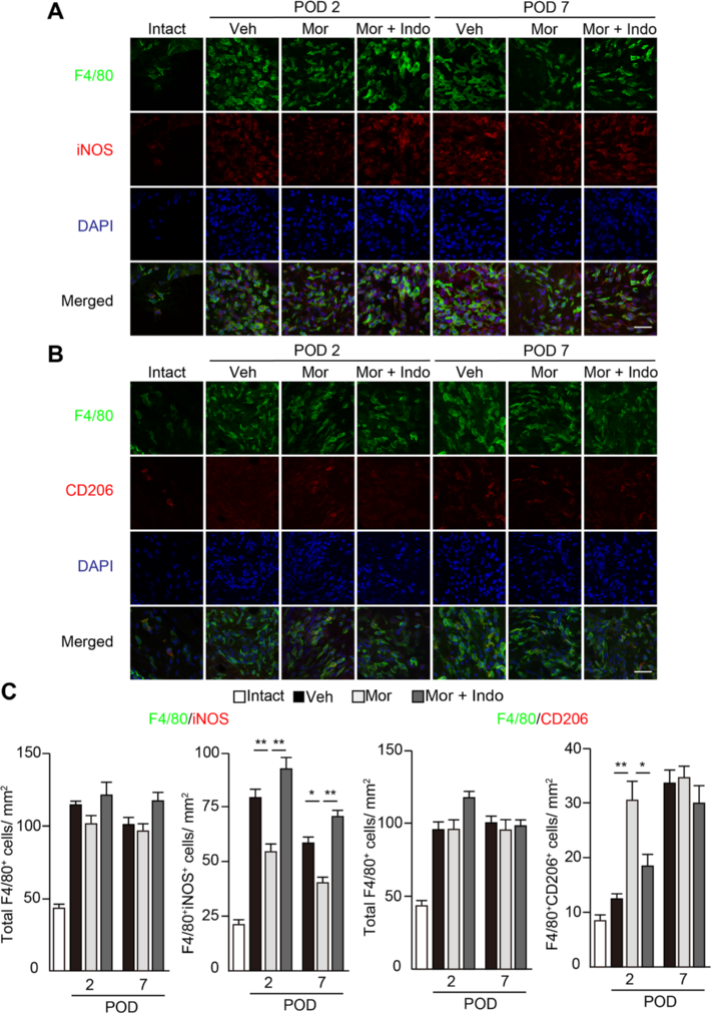
**Indomethacin reverses morphine-induced phenotype switch of macrophages.** Infiltration of F4/80^+^iNOS^+^ M1 macrophages **(A)** and F4/80^+^CD206^+^ M2 macrophages **(B)** were evaluated on PODs 2 and 7. **(C)** The numbers of total F4/80, F4/80^+^iNOS^+^, and F4/80^+^CD206^+^ macrophages were counted. Scale bar, 25 μm. *P < 0.05, **P < 0.01 (two-way ANOVA followed by Bonferroni *post-hoc* testing). Each column represents mean ± SEM (n = 6 for each group). Veh; vehicle (PBS + TB), Mor; morphine 10 μg, Indo; indomethacin 50 μg.

COX-2 inhibition by celecoxib promoted a phenotype shift of tumor-associated macrophages from M2 to M1 in a mouse model of colon cancer [28]. To evaluate the effects of COX-2 on macrophage polarization over the time course of the post-incisional process, the M1/M2 balance of macrophages at the incisional sites was evaluated by immunostaining with F4/80, DAPI and M1- and M2-specific markers, iNOS and CD206, respectively (Figure 8A and B). Coadministration of indomethacin blocked the reduction of F4/80^+^iNOS^+^ M1 macrophages induced by morphine on PODs 2 and 7 (Figure 8C, two-way ANOVA: F (2, 27) = 37.84, *P* < 0.0001), and reversed the increase in numbers of F4/80^+^CD206^+^ M2 macrophages on POD2 (Figure 8C, two-way ANOVA: F (2, 27) = 7.98, *P* = 0.002). Taken together with data showing indomethacin reversed morphine-induced analgesia (Figure 7A), local administration of morphine ameliorates mechanical hypersensitivity by altering the M1/M2 balance of macrophages via COX-2-dependent pathway.

We demonstrated that local administration of morphine in the early phase attenuated post-incisional mechanical hyperalgesia with a concomitant decrease in M1 macrophages and an increase in M2 macrophages. Depletion of macrophages, inhibition of HO-1 or COX all resulted in the reversal of morphine-induced analgesia, with an alteration in macrophage phenotype.

Previous studies have shown that infiltration of M1 macrophages preceded that of M2 macrophages during the course of pain development, resulting in the late onset of M2 macrophage-derived analgesic effects [5,6]. Although the phenotype shift of macrophages was observed on POD2 (Figure 3), morphine had no analgesic effects during PODs 1–5 (Figure 1A). This might be explained by the changes in the expression levels of IL-1β and enkephalin on POD7, but not POD2 (Figure 4). These data suggest that functional changes in macrophages require additional time after phenotype shift by the administration of morphine, which may also explain why levels of enkephalin were increased on POD7 (Figure 5) although the number of M2 macrophages was unchanged by morphine on POD7 (Figure 3). Another possibility is that the transient increase in pronociceptive PGE2 by morphine on POD2 (Figure 7B) may have resulted in a small analgesic effect in the early phase. Wolf *et.al.* reported that IL-1β-deficient mice exhibited complete abolishment of post-incisional pain behavior [27]. Intraplantar injection of small doses (0.1-100 pg) of IL-1β decreased the mechanical threshold [29]. Taken together with our data, IL-1β might be the critical mediator for the development of mechanical hyperalgesia, partly regulated by μ opioid receptor signaling (Figure 4).

Although it has been reported that a single administration of morphine into the hindpaw attenuated paw edema and thermal hyperalgesia in the acute phase of a carrageenan-induced inflammatory pain model [30], intraplantar morphine had no effect on paw edema when carrageenan was repeatedly administered [10]. Supporting these previous reports, in the present study peripherally administered morphine had no effect on paw edema or hyperalgesia to heat stimuli from 1 day after incision (Figure 1). We previously reported that a phenotype shift to M2 macrophages by PPARγ signaling altered the threshold to mechanical, but not thermal stimuli in a CFA-induced inflammatory pain model [6]. Thus, the development of thermal hyperalgesia may be modulated by macrophage-independent mechanisms.

In the present study, morphine was repeatedly administered for 3 days (days 0–2 or 5–7) after incision. Therefore, peripheral morphine tolerance is a concern for this study. Although the analgesic response was eliminated in mice receiving topical morphine alone for 3 days [31], Zollner *et al.* reported that mice receiving chronic morphine treatment (10 mg/kg subcutaneously twice daily for 4 days) did not develop tolerance at the peripheral μ-opioid receptors in the presence of CFA-induced paw inflammation [32]. Furthermore, they demonstrated that tolerance ensued when endogenous opioid peptides in inflamed tissue were removed by antibodies or by depleting opioid-producing leukocytes with cyclophosphamide. Because the majority of opioid-containing leukocytes were ED1^+^ monocytes/macrophages in the late stage of CFA–induced inflammation [21], the possibility that the phenotype of local macrophages might have played a role in peripheral opioid tolerance during the development of inflammatory pain could not be excluded. Further investigation is needed to clarify the involvement of macrophage polarization in opioid tolerance.

The expression of COX-2 was increased in the early phase by morphine administration, and the analgesic effects of morphine in the late phase were reversed by coadministration of indomethacin (Figure 7). The phenotypic shift of local macrophages by morphine was mediated by COX-dependent mechanism (Figure 8). COX-2/PGE2 is known to be a pronociceptive mediator mainly released by local macrophages during acute inflammation. In response to peripheral inflammatory challenges by the administration of carrageenan and CFA, mice lacking the ATP-gated P2X4 channel did not elicit pain hypersensitivity and lacked the COX-dependent release of PGE2 [25], suggesting that COX-dependent release of PGE2 from macrophages is essential for the development of inflammatory pain. Conversely, previous reports have demonstrated that PGE2 promoted the differentiation of macrophages to the anti-inflammatory M2 phenotype [33,34]. PGE2 release was enhanced from peritoneal macrophages isolated from morphine-dependent rats [27]. Although the expression levels of *Ptgs2* were not different between vehicle- and morphine-injected sites on POD7, morphine-injected mice exhibited less hypersensitivity to mechanical stimuli in the late phase. Therefore, we speculate that peripheral COX-2 in the microenvironment might have pronociceptive effects in the early phase, but inhibit the development of chronic pain by altering macrophage phenotype in the late phase.

**Figure 9:**
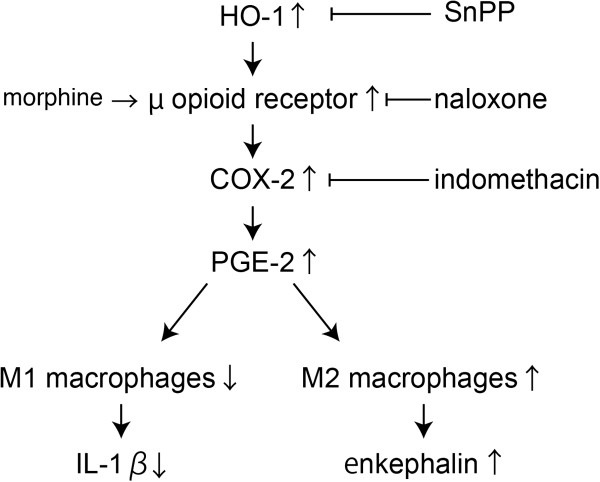
**Schematic diagram of morphine-induced analgesia representing the molecular pathways in macrophages.** Peripherally administered morphine alters macrophage polarity through a COX2/PGE2-dependent pathway. Morphine-induced changes in macrophage polarity decrease IL-1β levels, and increase PGE2 and enkephalin in the late phase. HO-1 might be upstream of μ opioid receptor signaling.

We previously reported that HO-1 induced macrophage polarity towards the M2 phenotype [6,9]. Despite our results showing HO-1 mRNA levels were unchanged by morphine treatment, the HO-1 inhibitor SnPP effectively reversed the effects of morphine on mechanical threshold and macrophage polarity (Figures 5 and 6). Hervera *et al.* reported that an HO-1 inducer, cobalt protoporphyrin IX increased μ opioid receptor protein levels in the ipsilateral dorsal root ganglia in a mouse model of neuropathic pain model [24], suggesting that μ opioid receptors might be downstream of the HO-1 signaling pathway. Because morphine increased the expression of PGE2, and the analgesic effects of μ opioid receptor were inhibited by a COX-2 inhibitor, indomethacin, the analgesic effects of μ opioid receptor signaling might be dependent on COX-2/PGE2 (Figure 9).

## Conclusions

We demonstrated that local administration of morphine attenuates the development of postincisional hyperalgesia through macrophage-dependent mechanisms. Phenotype shifts of local macrophages were induced by morphine administered early in the course of pain development, possibly through a COX2/PGE2-dependent pathway. Therefore, peripheral μ opioid receptors in macrophages could be a potential new therapeutic target for the development of postoperative pain therapies.

## Methods

### Animals

Male C57BL6 mice aged 8–10 weeks were obtained from CLEA Japan, Inc. (Tokyo, Japan). The Animal Research Committee of Kagoshima University approved all experimental procedures, which were implemented according to the guidelines of the National Institutes of Health and the International Association for the Study of Pain [35]. Mice were housed in groups of four or five per cage in a 12 hour light–dark cycle.

### Paw incision model

The mouse hindpaw plantar incision model was created as described previously [36]. Mice were deeply anesthetized by inhalation of 1.5–2.0% isoflurane (Abbott, Tokyo, Japan) via a nose cone. A 5-mm longitudinal incision was made with a No. 11 blade through the skin and fascia of the plantar foot. The incision was started 2 mm from the proximal edge of the heel and extended toward the toes. The underlying muscle was elevated with a curved forceps leaving the muscle origin and insertion intact. The skin was apposed with a single mattress suture of 8–0 nylon. Morphine (Shionogi & Co. LTD., Japan) was dissolved in phosphate-buffered saline (PBS, pH 7.2), SnPP (Tocris Bioscience, Bristol, UK) was diluted in dimethyl sulfoxide (DMSO), and indomethacin (Nacalai Tesque, Kyoto, Japan) was diluted in Tris buffer (TB, pH8.0). Morphine (3 μg/20 μL or 10 μg/20 μL), naloxone (5 μg/20 μL, Wako, Osaka, Japan), SnPP (400 nmol/20 μL) or indomethacin (50 μg/20 μL) were injected locally to the incisional sites 1 hour after the skin was sutured, and on PODs 1 and 2, or on PODs 5–7. The total amount of solution injected to the hind paws was 20 μL/paw for all experiments. The suture was removed at the end of POD2.

### Pain behavior

All behavioral experiments were performed by the same tester in a blinded manner. Withdrawal latencies to heat stimuli were assessed by applying a focused radiant heat source to an unrestrained mouse placed on a heat-tempered glass floor using the Paw Thermal Stimulator (UCSD, San Diego, CA, USA). A thermal stimulus was then applied to the plantar surface of each hind paw. Each mouse was tested at an interval of 2–3 minutes. The latencies to thermal stimuli were calculated as the mean of three trials. A cut-off time was set at 20.5 s to avoid tissue damage. To evaluate tactile allodynia, calibrated von Frey filaments (0.08–2.00 g) were applied to the plantar surface of the hindpaw from underneath the mesh floor. The 50% paw withdrawal threshold was determined using the updown method [37]. Behavioral experiments were performed before the administration of reagents to hind paws on PODs 1, 2, 5, 7, 10, 12, and 14.

### Measurement of paw edema

Post-incisional edema, reflected by an increase in dorsal-to-ventral paw thickness, was measured by a micro-caliper (Shinwa Measuring Tools; Niigata, Japan). The mean of three measurements at each time-point was used for analysis.

### Depletion of local macrophages

For macrophage depletion, 10 μL of clodronate encapsulated in liposomes (Clophosome-A) or empty control liposomes (Formu Max, Palo Alto, CA, USA) were locally injected into the incisional sites 1 hour after the skin was sutured, and on PODs 1 and 2.

### Immunohistochemistry

Mice were deeply anesthetized with sodium pentobarbital (50 mg/kg intraperitoneally) and transcardially perfused with saline. Tissues were fixed in 4% paraformaldehyde overnight at 4°C and placed in 30% sucrose solution for 24 h at 4°C. Sections (30 μm thick) were incubated overnight with primary antibodies to pan-macrophage marker, F4/80 (1:100; Santa Cruz Biotechnology, Santa Cruz, CA, USA), iNOS (1:500; Abcam, Cambridge, UK), or CD206 (1:100; Santa Cruz Biotechnology) at 4°C overnight and then incubated for 1 hour at room temperature with Alexa Fluor 488- or Alexa Fluor 546-conjugated secondary antibody (1:500; Invitrogen, Carlsbad, CA, USA) followed by nuclear staining with DAPI (Vector Laboratories, Burlingame, CA, USA). Fluorescent images were obtained using an LSM700 imaging system (Carl Zeiss, Aalen, Germany). The intensity of F4/80 immunofluorescence at clodronate-treated sites, the number of total F4/80^+^, F4/80^+^iNOS^+^, or F4/80^+^CD206^+^ cells with clearly visible nuclei stained by DAPI were evaluated using Image J 1.43u 2010 software (National Institutes of Health, Bethesda, MD, USA).

### Quantitative PCR

Total RNA of hind paws was extracted from the hindpaw using Sepazol reagent (Nacalai Tesque, Kyoto, Japan). The synthesis of first-strand cDNA was performed using High Capacity RNA-to-cDNA (Applied Biosystems, Carlsbad, CA, USA) according to the manufacturer’s instructions. Quantitative PCR was performed on an ABI Prism StepOnePlus real-time PCR System (Applied Biosystems) TaqMan assays were performed for quantification of *Il-1β* (assay ID:Mm00434228_m1), *Tnf* (assay ID:Mm00443260_g1), *Penk* (assay ID:Mm012128758_m1), and *Hmox-1* (assay ID:Mm00516005_m1) using TaqMan Fast Advanced Master Mix (Applied Biosystems) according to the manufacturer’s instructions. Target gene expression was normalized to glyceraldehyde 3-phosphate dehydrogenase.

### Statistical analysis

Data are presented as mean ± SEM. Differences among groups were analyzed using one-way or two-way ANOVA followed by Bonferroni *post hoc* testing (Graphpad Prism 5.0, La Jolla, CA, USA). A value of *P* < 0.05 was considered significant.

## Abbreviations

ANOVA: Analysis of variance; CCR2: C-C chemokine receptor 2; CFA: Complete Freund’s adjuvant; COX: Cyclooxygenase; DMSO: Dimethyl sulfoxide; HO: Heme oxygenase; IL: Interleukin; iNOS: Inducible nitric oxide synthase; PBS: Phosphate-buffered saline; Penk: Preproenkephalin; PGE2: Prostaglandin E2; POD: Postoperative day; PPARγ: Peroxisome proliferators-activated receptor γ; Ptgs2: Prostaglandin-endoperoxide synthase 2; SnPP: Tin protoporphyrin; TB: Tris buffer; TNF: Tumor necrosis factor.

## Competing interests

The authors declare that they have no competing interests.

## Authors’ contributions

All authors read and approved the final manuscript. MH-M and YK designed the study. KG, TK, TSai, TY, TSat and MK performed the experiments. KG and MH-M wrote the manuscript.
